# Employees and the National Insurance Act

**Published:** 1912-03-23

**Authors:** 


### NATIONAL INSURANCE ACT DEPARTMENT.

### EMPLOYEES AND THE NATIONAL INSURANCE ACT.

### III.—Benefits Receivable under the Act.

We proceed this week to give an explanation of the benefits, including donation benefits, which the Act contemplates. It is intended in this National Insurance Act Department to deal with the benefits receivable under the Act as they apply to different classes of persons, more particularly with the staffs, both male and female, of voluntary hospitals and kindred institutions, and also with that large body of men and women workers of the artisan class who, of late years, have so generously supported the voluntary hospitals. Before dealing with the benefits as applicable to special classes of persons it is necessary to describe the standard benefits which form the basis of the Act.

#### Standard Benefits.

These benefits are: (*a*) Medical benefit, (*b*) Sanatorium benefit, (*c*) Sickness benefit, (*d*) Disablement benefit, (*e*) Maternity benefit, and (*f*) Additional benefits. These benefits apply equally to all insured persons in Great Britain who are members of approved societies either as compulsory or voluntary contributors. They also apply, with the exception of the additional benefits, to deposit contributors. In this issue particulars are set forth as to the first two of the benefits included in the above list. Next week we shall deal with sickness and disablement benefits, reserving to further issues a description of the maternity and additional benefits.

#### (*a*) Medical Benefit.

This is defined by the Act as "Medical treatment and attendance, including the provision of proper and sufficient medicines, and such medical and surgical appliances as may be prescribed by regulations to be made by the Insurance Commissioners."

It is to be administered by the Insurance Committee of the county or county borough in which the insured member resides. The Insurance Committee is required to make arrangements with duly qualified medical practitioners to secure adequate medical attendance and treatment. Provision must also be made by the Committee for the supply of proper and sufficient drugs and medicines, and prescribed appliances to insured persons from chemists and druggists, with whom arrangements for such supply have been made, if ordered by the doctor in attendance.

#### Free Medical Attendance.

A list, called a panel, of the medical practitioners who have agreed to attend and treat insured persons, must be prepared and published, and every insured person is to have the right of selecting from the list applicable to his county or county borough the doctor by whom he wishes to be attended. If the doctor consents to the selection the insured person will have the right to his attendance and treatment, free of cost to himself, whenever his services are required.

The cost of this benefit is estimated, in the calculations on which the contributions (including that of the State) are based, at 6s. per head per annum, or about 1½d. per week. This is not, however, a fixed and unalterable allowance, and is in fact not mentioned specifically in the Act. It will doubtless form the basis of the agreements to be made between the approved societies and the local Insurance Committees as to the administration of the benefit, but in the event of the doctors requiring a higher remuneration and thus placing the Committee in the position of having a larger estimated expenditure than it has estimated income attributable to medical benefit, such Committee may take steps to obtain the sanction of the Treasury and of the Council of the local county or county borough to such additional expenditure. If this sanction be granted by both authorities they shall each bear one-half of the sums so sanctioned.

Controversy has been rife as to the adequacy of the remuneration to the doctors attending patients under the State scheme, and as to the conditions of their services, but this does not concern us directly in the consideration of the medical benefit itself.

#### The Income Limit.

There is, however, one matter arising out of this controversy which will probably affect a large number of insured persons, and which must therefore be considered. This is in reference to the income limit of those entitled to medical benefit.

By the general provisions of the Act employed persons with incomes not greater than £160 per annum are compulsorily insured, but for the purpose of medical benefit it is laid down, as an outcome of the controversy alluded to, that every Insurance Committee has the power to fix a limit of income for its own area, and to require that all insured persons in receipt of an income exceeding that limit shall make their own arrangements for receiving medical attendance and treatment, including medicines and appliances. Other persons with incomes below the prescribed limit may be allowed to make their own individual arrangements. In such cases the Committee shall contribute from the funds out of which medical benefit is payable towards the cost of medical treatment, such sums as shall not in total exceed the amounts which the Committee would otherwise have expended in providing medical benefit for them. The contention of the doctors is for a £2 per week income limit, and if this be agreed to by the Insurance Committees—on which, by the way, the doctors are specially represented—the free medical attendance, whenever required, will be modified in an important degree.

No person will be entitled to medical benefit during the first six months after the Act comes into operation. Thus the earliest date on which it will be possible for the benefit to be received will be January 15, 1913.

It should also be noted that an insured person in Ireland is not entitled to medical benefit, but as an offset against this reduction the joint weekly contribution of the employer and employee is reduced by 1½d. both for men and women.

Advantage to the Insured Person.

There is no doubt that the free provision of adequate medical attendance and treatment will be an inestimable boon to a large section of the community who have been in the habit of deferring attention to minor ailments, which could have been readily cured at an early stage, until their health has become seriously impaired and their earning capacity reduced in consequence. In certain classes of employment provision of medical attendance has commonly been made by the employer even if it has not been expressly guaranteed as a condition of such employment. In all such cases there will be no further necessity for the employer to provide the attendance, and the employee will have the right of selecting the particular doctor by whom he wishes to be treated. Alternative benefits are not allowed in substitution for or in reduction of the medical benefit.

(*b*) Sanatorium Benefit.

This is defined by the Act as "Treatment in sanatoria or other institutions or otherwise when suffering from tuberculosis, or such other diseases as the Local Government Board with the approval of the Treasury may appoint." Like medical benefit this is to be administered by the Insurance Committees, which are required to make satisfactory arrangements for the treatment of persons suffering from tuberculosis, and other prescribed diseases, with persons or local authorities (other than Poor Law authorities) having the management of sanatoria or other institutions approved by the Local Government Board.

It is to be noted that the sanatorium or other institution in which the insured person is treated need not be situated in the area in which he resides, and for this reason power is given to local authorities, if so authorised by the Local Government Board, to provide treatment as respects insured persons resident outside as well as those resident within their area. This is an important provision, because it is manifest that in many thickly populated areas the provision of sanatoria would not only be prohibitive on the ground of expense, but also on account of the unsuitability of the situation.

Provision for Treatment of Dependants.

Though primarily designed for the benefit of the insured persons, the benefit may be extended to the dependants of the insured persons, or to any class of such dependants as are resident in the county, or any part of the county, or in the county borough, if the Insurance Committee thinks fit. The extension of the benefit to the dependants of insured persons was not provided in the Bill when first presented to Parliament, but was inserted during the progress of the measure through the House. It will be seen that the amendment is of a far-reaching character, and if the Insurance Committees see fit to extend the benefit in the manner indicated it will be much more effective in its operation than in its original form.

The sums available for defraying the cost of sanatorium benefit are 1s. 3d. per annum, to be provided in respect of each insured person out of the joint contributions of the employer and employee, together with a further sum of 1d. per annum in respect of each such person to be payable out of moneys provided by Parliament. No person is to be entitled to sanatorium benefit unless the Insurance Committee recommends the case for such benefit.

As distinguished from medical benefit there is no specific waiting period after the commencement of the Act which must elapse before sanatorium benefit comes into operation. It is, of course, apparent that a very large number of insured persons will contribute their quota year after year, so long as they remain insured persons, towards the sanatorium benefit, and never receive any direct personal benefit therefrom. The scourge of consumption is, however, a National evil, and its treatment on National lines was a fundamental in a scheme for National health insurance.

Erection of Sanatoria.

Very closely allied to the administration of sanatorium benefit are the provisions of the Act relating to the erection and maintenance of sanatoria and similar institutions. It is proposed to make a special grant of £1,500,000 during the present session of Parliament towards the erection of such sanatoria, and this sum or any other that may be voted for the purpose is to be divided between England, Wales, Scotland, and Ireland in proportion to their respective populations as disclosed by the 1911 census. Such sum as may be divided will be a substantial grant-in-aid towards the initial cost of the buildings.

As an outcome of this special legislation it is to be hoped that the design of its author to stamp out, if possible, the dreaded scourge of consumption may be successfully accomplished.

[NOTE.—Questions and correspondence on all doubtful points are invited and will be answered in this column.]

